# A Retrospective Study of 158 Cases on the Risk Factors for Recurrence in Ameloblastoma

**DOI:** 10.7150/ijms.61500

**Published:** 2021-07-25

**Authors:** Ling Bi, Dong Wei, Dongsheng Hong, Jin Wang, Kejia Qian, Huiming Wang, Huiyong Zhu

**Affiliations:** 1 Department of Stomatology, The First Affiliated Hospital, Zhejiang University School of Medicine , Hangzhou, 310003,China; 2 Key Laboratory for Drug Evaluation and Clinical Research of Zhejiang Province, The First Affiliated Hospital, Zhejiang University School of Medicine, Hangzhou, 310003,China; 3 The Affiliated Hospital of Stomatology, School of Stomatology, Zhejiang University School of Medicine, and Key Laboratory of Oral Biomedical Research of Zhejiang Province, Hangzhou, Zhejiang, 310006, China

**Keywords:** ameloblastoma, risk factor, recurrence, survival analysis

## Abstract

**Background:** Ameloblastoma is an odontogenic tumor occurring in jaws, with local aggressiveness and postoperative recurrence. This study was aim to investigate the clinical and radiographic risk factors for recurrence in ameloblastoma.

**Methods:** Patients diagnosed with ameloblastoma between March 2009 and March 2019 were retrospectively analyzed. Clinical and Radiological data and follow-up records were collected. Survival analyses were performed by Kaplan-Meier and log-rank tests, as well as Cox proportional hazards model.

**Results:** One hundred and fifty-eight patients (104 males and 54 females were enrolled. The overall recurrence rate for ameloblastoma was 13.29%, and 10.76% recurred within 5 years. Most of the tumors were located in mandible (86.71%), while the rest 21 cases were in maxilla (13.29%). More than half cases (55.06%) showed multilocular radiolucency, 61 cases (38.61%) showed unilocular radiolucency. Significant differences were found with amelobastoma recurrence rate related to treatment modality, impacted tooth and root resorption (P =0.002, 0.022 and 0.007 respectively).

**Conclusions:** Treatment modality, impacted tooth and root resorption all showed statistically significant associations with the recurrence rate in ameloblastoma. However, due to the limitation of this study, further studies are needed to reveal the true mechanism of ameloblastoma recurrence.

## Introduction

Ameloblastoma is a benign tumor of epithelial origin with mature, fibrous stroma but without odontogenic ectomesenchyme.^1^ It occurs in the upper and lower jaws. About 80% of ameloblastomas occur in mandible, and the remaining 20% are found in maxilla. Overall, it accounts for 9% to 11% of human odontogenic tumors.^2^ According to the newly updated classification of the World Health Organization (WHO) in 2017, ameloblastoma is classified into four subtypes: conventional ameloblastoma, unicystic ameloblastoma, peripheral/extraosseous ameloblastoma and metastasizing ameloblastoma.^3^

Although ameloblastoma is benign odontogenic tumor of jawbones, it is locally aggressive with a high postoperative recurrence rate up to 55%-90%.^4^-^6^ Also a few rare cases of distant metastatic ameloblastomas were reported.^7^, ^8^ The growth or relapse of ameloblastoma causes functional and cosmetic hazards, such as jaw swelling, teeth loosing, shifting and shedding. It leads to facial deformities and dysfunction. In particular, those occurring in the maxilla could invade the maxillary sinus and spread to the orbit and the nasolacrimal duct, causing the eyeball shifting, protruding, tearing and diplopia. Complete resection is still the primary means of reducing recurrence at present.^9^

In order to reduce the recurrence of ameloblastoma and to minimize the impact of repeated surgery on patients' facial appearance and function, it is important to identify the risk factors associated with recurrence. It is also useful for clinical treatment protocol and prognosis judgement. Its aim was to investigate the clinical and radiographic risk factors associated with recurrence in ameloblastoma over a decade.

## Materials and methods

### Study design and data collecting

This respective study was in compliance with Helsinki Declaration. It was approved by Clinical Research Ethics Committee of the First Affiliated Hospital, College of Medicine, Zhejiang University (Trial registration: ChiCTR2000041196). All the patients were treated in the Department of Oral and Maxillofacial Surgery of the hospital.

According to the patients' files, CT and X-ray data, patients diagnosed with ameloblastoma were selected during the decade of 2009 to 2019. Inclusion Criteria: (1) confirmation of ameloblastoma histological diagnosis according to the WHO recommendation, (2) cases diagnosed for the first time between March 2009 and March 2019, (3) follow-up for more than 1 year, (4) follow-up cases that could be tracked in the electronic case system.

Among the available patients' records the data was collected including: (1) Sex: male or female. (2) Age, including three groups: ≤30 years old, 31-50 years old, and ≥51 years old. (3) Tumor site, mainly considered two different locations: maxilla and mandible. And tumor subsites, according anterior and posterior regions. (4) Treatment, grouped into enucleation only and resection. Several cases of both groups were received preoperative marsupialization. (5) WHO classification, including four types: conventional ameloblastoma, unicystic ameloblastoma, peripheral/extraosseous and metastasizing ameloblastoma. (6) Radiographic patters: monocystic type, multilocular type and others. (7) Other radiographic findings: invasion of cortical bone, impacted tooth involvement, tooth resorption, and pathological fracture. All the radiological features were obtained from either panoramic or cone Beam CT, combined with intraoperative findings. (8) Presence of soft tissue infiltration. (9) Follow-up, dating from first histological diagnosis to the last follow-up. (10) Recurrence, including the interval dating from the first treatment to the first relapse confirmed histopathologically.

### Statistical analysis

Percentages were used to express categorical variables and mean ± standard deviation was for Continuous variables. The survival function was evaluated by the Kaplan-Meier method. Survival differences between groups were compared by Log-rank test. Univariate and multivariate Cox proportional hazards model were used to analyze the prognostic factors. A P-value less than 0.05 was considered to be statistically significant. All of the statistical analyses were performed using SPSS19.0 for Windows (SPSS, Inc., Chicago, IL) R version 3.5.1 (R Core Team) was used for the drawing of all graphics.

## Results

One hundred and fifty-eight ameloblastoma patients were selected in the research institute during the decade of 2009 to 2019. Twenty-one patients were confirmed with first recurrence in ameloblastoma.

### Demographic and Clinical Data of All Eligible Patients

The statistical results of patient demographics, tumor sites, treatment modalities, and WHO classification were shown in Table 1.

#### Patient demographics

A total of 158 patients were involved. There were 104 male patients and 54 female patients (1.93: 1). Only 26 patients (16.46%) were over the age of 50, while 68 patients (43.04%) were 31-50 years old and 64 patients (40.50%) were in the group of ≤30 years. The follow-up period ranged from 14 to 132 months (mean 65 months, median 64 months).

#### Tumor site

Most of the tumors were located in the mandible (86.71%), with 128 cases in posterior region and seven in anterior region. And the rest 21 were in the maxilla (13.29%), with 20 cases in posterior region (Table 1, Fig.2A). Three patients had tumors extending through anterior and posterior regions, including one case in maxilla and two in mandible. While the rest patients had tumors in one region of mandible or maxilla.

#### WHO classification

97 cases (61.39%) were diagnosed as conventional ameloblastoma. 60 cases (37.93%) were unicystic ameloblastoma. Peripheral ameloblastoma was confirmed in only one case (0.63%). According to pathology diagnosis, tumor cells in this case were found only in the gingival, not in the adjacent bone tissue. No case was recorded as metastasizing ameloblastoma.

#### Treatment modalities

107 cases (67.72%) were treated with radical resection including partial or total removal of the jaw. In these cases, 75 were conventional ameloblastomas and 31 were unicystic ameloblastomas. Enucleation was used in 51 cases (32.28%). Of the total enucleation cases, 22 were conventional ameloblastomas and 29 were unicystic ameloblastomas. Fenestration was performed in one case during the operation and in the other eight cases preoperatively.

### Radiological and other characteristics

The statistical results of radiological and other characteristics were shown in Table 2.

More than half cases (55.06%) showed multilocular radiolucency, 61 cases (38.61%) showed unilocular radiolucency. Only one case (0.63%) showed no radiographic radiolucency and this radiographic finding was observed in the only peripheral ameloblastoma case, as expected. The other nine cases (5.70%) showed mixed radiolucent-radiopaque feature.

No soft tissue infiltration occurred in most cases (93.67%). The impacted tooth was involved with the tumor in 94 cases (59.49%). The third mandibular molar was the most common impacted tooth. Root resorption (87.97%) and cortical bone invasion (98.10%) were both very common. No pathological fracture was reported in any case.

### Follow-up of patients and analysis of prognostic factors for recurrence

**Fig 1:**
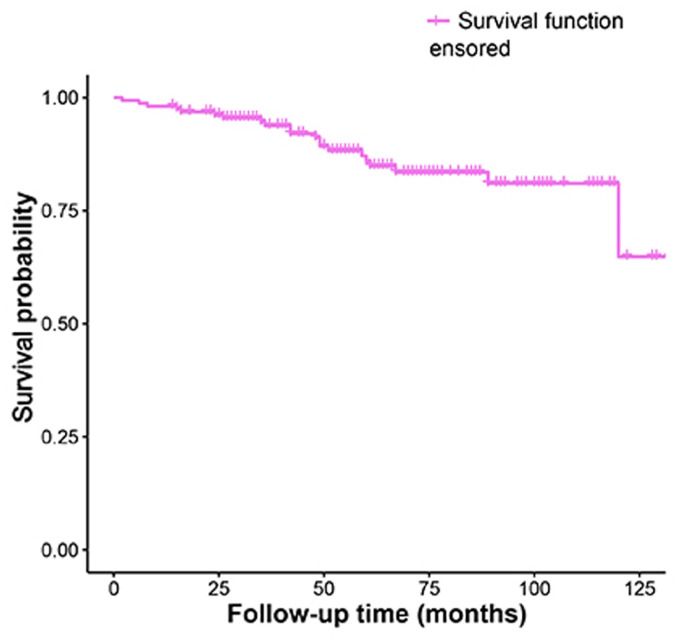
Kaplan-Meier survival funciton curve for recurrence time.

The Kaplan-Meier analysis of recurrence time was shown in Fig. 1.

**Fig 2:**
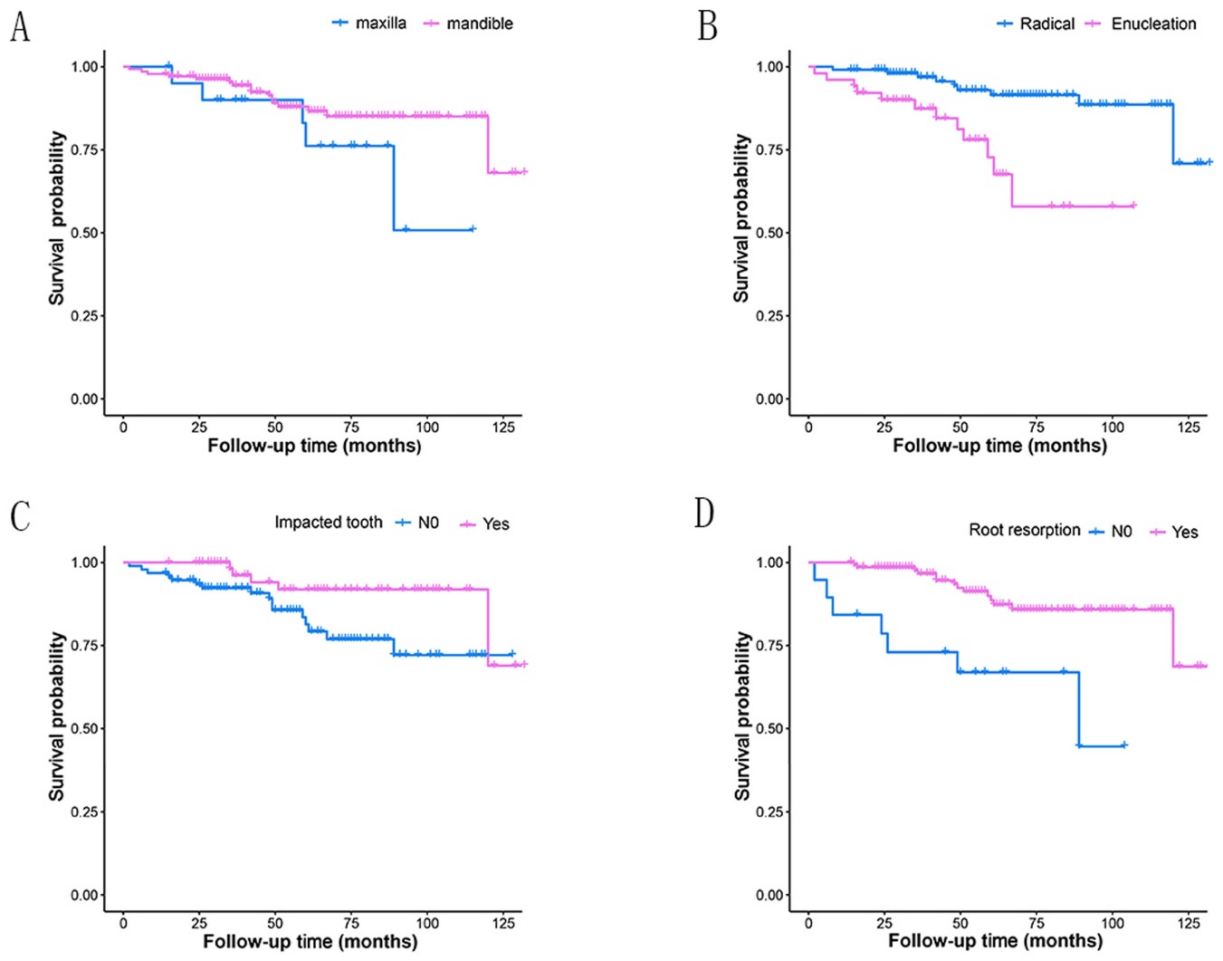
Kaplan-Meier survival curves for different groups depending on four prognostic factors. A: tumor site(P=0.130); B: treatment modality(P<0.001); C: impacted tooth(P=0.034); D: root resorption(P<0.001).

Treatment modality were significantly associated with recurrence (P=0.001) (Table 1) and the Kaplan-Meier curves for the two different treatment methods were presented in Fig. 2B. The recurrence risk of cases treated with enucleation was 4.62 times that of those treated with radical treatment. Statistically significant associations with recurrence were also revealed in the factors of impacted tooth and root resorption (Table 2, Fig.2C and 2D). Tumors with impacted tooth had areduced risk ratio (65.4%) of recurring compared to those without (HR 0.35, 95% CI 0.12-0.96, P=0.042). Similarly, more than three-quarter of the risk ratio in tumors with root resorption (77.5%) was reduced (HR 0.23, 95% CI 0.09-0.57, P=0.002). Furthermore, Table 3 shows the HRs of amelobastoma recurrence rates related to treatment modality, impacted tooth and root resorption according to multivariate Cox proportional hazards models. Significant statistical differences still existed, with the P-values were 0.002, 0.022 and 0.007, respectively.

Recurrence rates did not differ significantly among the three age groups. The highest (19.12%) rate was in the age group of 31-50 years old (Table 1). The other variables showed no statistically significant relationship with recurrence either, including sex, site, WHO classification, radiographic pattern, cortical bone invasion and soft tissue infiltration. Without significance, tumors in mandible had an approximately half reduction (53%) of recurrent ratio compared to those in maxilla (HR 0.47, 95% CI 0.17-1.29, P=0.143). And among those subsites, the recurrence rate in maxillary posterior region is relatively higher than others, but without any significant difference.

## Discussion

The total recurrence rate of ameloblastoma was 13.29% in this study, and 10.76% recurred within 5 years. 2.53% cases had recurrences twice. Significant differences were found when comparing recurrence rates in relation to treatment modality, impacted tooth and root resorption. It is worth mentioning that the association to the histological patterns and ameloblastoma recurrence also might be very important. However, due to the incomplete information of histological patterns in this study, we did not analyze it at present. Thus, this may be defined and investigated in further research.

The recurrence rate is identical to that reported by Siar et al.^10^, and also within the range of 9.78% to 20.6% showed by others^11^-^13^. Ameloblastoma seemed to be more common in males and the male-to-female ratio of our study (1.93:1) was higher than that of some previous studies (between 1.03:1 and 1.4:1).^9^, ^14^-^17^ Similar to other previous studies, the age of morbidity in this study ranged from 8 to 80 years.^9^,^10^,^14^,^15^ So referring to these similar studies, we chose two age cut-off points, 30 and 50, and established three age groups relatively evenly. Patients under 50 years old were more prone to recur than those over 50 years old. According to the WHO classification of ameloblastoma, 5 to 15% of all ameloblastomas are of the unicystic type.^18^ However, our study showed a higher proportion for 37.97% which was consistent with 38.3% reported in a previous study.^9^

Our study showed that 86.71% of ameloblastomas occurred in the mandible. It was similar to the results reported in other studies.^12^, ^19^ The mandible-to-maxilla ratio in our study was 6.52:1. It was lower than those in some systematic studies with large sample sizes, some of which were as high as 10.78:1.^20^, ^21^ During our follow-up period, although there was no significant difference in recurrence rate between different lesion sites, maxilla origin ameloblastoma had a higher tendency to relapse (Fig. 2A). Tumor cells could extend beyond the radiographic margin in cancellous bone at an average of 4.5 mm, even up to 8 mm.^22^ Since the cortical bone of maxilla is thinner than that of mandible, it is easier for tumor cells to infiltrate into cortical bone and, even earlier, to extend into adjacent soft tissue.^22^ This may be considered one of the important reasons why ameloblastoma in maxilla is more prone to recur. Compared to enucleation in this study, radical resection was confirmed to be more effective for controlling the recurrence rate of ameloblastoma (p<0.01, Fig.2B). This was also in line with the results of many other studies, including a couple of systematic reviews and meta-analyses.^23^-^25^ As mentioned earlier, the tumor cells may extend beyond the radiolucent margin by nearly as much as 10 mm^22^, so it would be very difficult to identify the true margin of the tumor, both clinically and radiologically. Therefore, the best method to reduce the recurrence of ameloblastoma was considered to be radical resection marginally or segmentally with safety margins.^26^ This was particularly important for maxillary ameloblastoma, which manifested more aggressive behavior, and for some histologically more aggressive subtypes like desmoplastic conventional ameloblastoma.^22^, ^25^ Interestingly, based on the WHO classification, the recurrence rate of unicystic ameloblastomas was higher than that of conventional ones. In our study, there were 97 cases of conventional ameloblastoma. Among them, 75 cases (77.32%) underwent radical resection. However, only 22 out of 51 unicystic cases (43.14%) were treated with radical resection. A higher proportion of conventional ameloblastoma cases with radical resection might be one of the key factors. Consistent result was also obtained based on radiographic pattern. The recurrence rate of unilocular ameoblastomas was higher than multilocular ones. Au et al. reported similar result and considered that those lesions of unilocular ameoblastomas were treated more conservatively.^9^ This might be close to the view that unicystic amloblastoma had a better prognosis, so it was often treated more conservatively.^27^

In many cases of this study, impacted tooth and root resorption were recorded. But few previous studies have shown that these two factors have a significant impact on recurrence rate. The novelty was that the present study found a significant correlation between these two factors and tumor recurrence. The reason for this remains unclear and may be controversial. Considering lower recurrence rate in cases with impacted teeth, a possible explanation is that mandibular posterior area is the most common location for both ameloblastoma and impacted third molar.^14^, ^28^ And this study showed that only three of the 21 maxillary cases had impacted teeth. But it occurred in nearly half of the 137 mandibular cases. An impacted tooth, like third molars in the mandible, has epithelial components with the potential for developing diverse neoplasms and lesions, such as the ameloblastoma. Thus, as an aetiologic factor, when removing adequately the developed neoplasm, the impacted tooth and its histological components, it is unlikely that a secondary lesion may arise from the first. This is also consistent with that the mandibular ameloblastomas have a lower recurrence rate than maxillary ones.^22^ Another possible reason is that the proportion of impacted teeth (40.51%) in this study is higher than those reported by other researchers 0-26.0%.^14^ Meanwhile, the root resorption rate (87.97%) is much higher than that in other studies^9^, ^28^. This may be due to a difference in judgement on radiological imaging between different researchers. Depending on the quality of image during the different follow-up periods, some critical states of root resorption are discovered by some researchers. Therefore, it is necessary to define a criterion for root resorption. In fact, for cases with root resorption in this study, surgeries tended to implement more thorough treatment. It might be one possible reason for lower recurrence rate of them than that of cases without root resorption.

However, the choice of the method for primary treatment will be affected by many factors. For children and adolescents, the maxillofacial dysfunction and deformity caused by the radical resection can have a negative impact on their future physical and psychological development.^29^ In order to minimize the postoperative complications and recurrence rate for pediatric group, the clinicians need to make the most appropriate surgical decision based on histological type by preoperative or intraoperative biopsy and tumor size.^29^ Even in some adult cases, extensive removal of tumors could have caused facial deformities and dysfunctions, or pathological fractures.^24^ So for some cases, such as huge mandibular cystic ameloblastoma, Marsupialization combined with second-stage surgical resection is recommended as primary treatment method.^30^ Of course, the implementation of aesthetic and functional rehabilitation is also necessary and could be performed at the same time or second-stage of resection.^24^, ^31^ In addition, the treatment decision making is influenced by the patient's family economic situation and different resources of health care system.^21^, ^31^

In conclusion, treatment modality, impacted tooth and root resorption all showed statistically significant correlation with recurrence in ameloblastoma. However, there are still some limitations in this study, including small sample size and insufficient follow-up period relatively. The real role of impacted tooth and root resorption in ameloblastoma recurrence needs to be further investigated by more studies.

## Figures and Tables

**Table 1:** Patient demographics, tumor sites, treatment modalities, and WHO classification.

Variables and Categories	Total number	Recurrence number (%)	HR	95%CI	P-value
Sex					
Male	104	14(13.46)	0.9	0.36-2.25	0.814
Female	54	7(12.96)	Ref		
Age(years)					
≤30	64	6(9.38)	Ref		
31-50	68	13(19.12)	2.15	0.81-5.69	0.124
≥51	26	2(7.69)	0.79	0.16-3.92	0.773
Site					
Maxilla	21	5(23.81)	Ref		
Mandible	137	16(11.68)	0.47	0.17-1.29	0.143
Subsite					
Posterior maxilla	20	5(25.00)	1.01	0.59-1.7	0.97
Anterior mandible	7	1( 14.29)	1.31	0.57-3.0	0.53
Posterior mandible	128	15(11.72)	Ref		
Anterior + Posterior maxilla	1	0(0)			
Anterior + Posterior mandible	2	0(0)			
Treatment					
Radical treatment	107	9(8.41)	Ref		
Enucleation	51	12(23.53)	4.62	1.84-11.62	0.001
WHO classification					
Conventional ameloblastoma	97	12(12.37)	Ref		
Unicystic ameloblastoma	60	9(15.00)	1.75	0.73-4.18	0.210
Peripheral ameloblastoma	1	0(0)			
Metastasizing ameloblastoma	0				

WHO, World Health Organization; HR, hazard ratio; CI, confidence interval; Ref, reference category.

**Table 2:** Radiological and other characteristics.

Variables and Categories	Total number	Recurrence number (%)	HR	95%CI	P-value
Radiographic pattern					
Multilocular	87	9(10.34)	Ref		
Unilocular	61	9(14.75)	1.78	0.69-4.63	0.235
Other (mixed radiolucent-radiopaque)	10	3(30.00)	2.61	0.69-9.86	0.157
Impacted tooth					
No	94	16(17.02)	Ref		
Yes	64	5(7.81)	0.35	0.12-0.96	0.042
Root resorption					
No	19	7(36.84)	Ref		
Yes	139	14(10.07)	0.23	0.09-0.57	0.002
Cortical bone invasion					
No	3	1(33.33)	Ref		
Yes	155	20(12.90)	0.25	0.03-1.86	0.175
Soft tissue infiltration					
No	148	19(12.84)	Ref		
Yes	10	2(20.00)	1.54	0.36-6.66	0.565

**Table 3:** Multivariate cox analysis of three risk factors.

Variables and Categories	HR	95% CI	P-value
Treatment			
Radical treatment	Ref		
Enucleation	4.42	1.72-11.35	0.002
Impacted tooth			
No	0.30	0.11-0.84	0.022
Yes	Ref		
Root resorption			
No	0.28	0.11 -0.70	0.007
Yes	Ref		

## Abbreviations

WHO: World Health Organization
HR: hazard ratio
CI: confidence interval
Ref: reference category
